# Metabolic Dysfunction-Associated Fatty Liver Disease in the National Health and Nutrition Examination Survey 2017–2020: Epidemiology, Clinical Correlates, and the Role of Diagnostic Scores

**DOI:** 10.3390/metabo12111070

**Published:** 2022-11-05

**Authors:** Panagiotis Theofilis, Aikaterini Vordoni, Rigas G. Kalaitzidis

**Affiliations:** General Hospital of Nikaia-Piraeus Agios Panteleimon, Center for Nephrology “G. Papadakis”, 18454 Piraeus, Greece

**Keywords:** metabolic dysfunction-associated fatty liver disease, epidemiology, risk factors, fatty liver index, liver fibrosis

## Abstract

The recent establishment of metabolic dysfunction-associated fatty liver disease (MAFLD) has led to a reevaluation of its epidemiology, diagnosis, and clinical implications. In this study, we aimed to evaluate MAFLD’s epidemiology and its association with other pathologic states and biomarkers, as well as to assess the prevalence of the different fibrosis stages in the MAFLD population, together with the importance of diagnostic scores in the preliminary determination of significant fibrosis. After analyzing the National Health and Nutrition Examination Survey (NHANES) 2017–2020, we found a high prevalence of MAFLD, at 58.6% of the studied population. MAFLD was accompanied by numerous comorbidities, which were increasingly common in individuals with higher grades of liver fibrosis. Fatty liver index emerged as a reliable indicator of MAFLD, as well as significant fibrosis. The estimation of fatty liver index could be a reasonable addition to the evaluation of patients with metabolic risk factors and could lead a diagnosis in the absence of liver elastography or biopsy. Further studies are needed to enhance our knowledge regarding its prognosis, as well as the role of novel therapies in its prevention or regression.

## 1. Introduction

Obesity and type 2 diabetes mellitus (T2DM) are two metabolic disorders that are modern pandemics with high prevalence and escalating incidence [1,2]. Even though cardiovascular diseases are the most common complication, liver injury is increasingly frequent, as shown by lately published epidemiologic trends of non-alcoholic fatty liver disease (NAFLD) and non-alcoholic steatohepatitis (NASH) [3,4]. However, the existing NAFLD definition is based on exclusion criteria. This inconvenience was addressed by a recent expert consensus that provided a concise and thorough definition and diagnostic criteria for the so-called metabolic dysfunction-associated fatty liver disease (MAFLD) [5].

The recent establishment of MAFLD as an entity underscores the importance of strenuous research to better define the epidemiological trends and clinical spectrum. Until recently, the vast majority of research on fatty liver disease was confined to NAFLD. However, studies have shown that MAFLD may better predict the risk of cardiovascular disease in asymptomatic individuals compared to NAFLD [6]. Unfortunately, since the research on MAFLD is just beginning, evidence is scarce regarding the incidence and prevalence of the disease in the general population, as well as in specific subpopulations. Moreover, its prospective association with pathologic states is poorly understood due to the lack of big data. Finally, as liver fibrosis may be an even stronger cardiovascular risk factor [7], the use of scores could represent an alternative to its efficient identification, since the more credible methods may be either invasive (liver biopsy) or not readily available (transient elastography) [8].

Considering all of the above, this analysis of the National Health and Nutrition Examination Survey (NHANES) 2017–2020 aims to delineate MAFLD’s epidemiology and its association with other pathologic states and biomarkers. Moreover, we sought to evaluate the prevalence of the different fibrosis stages in the MAFLD population, together with the importance of diagnostic scores in the preliminary determination of significant fibrosis.

## 2. Materials and Methods

### 2.1. Study Population

**Figure 1:**
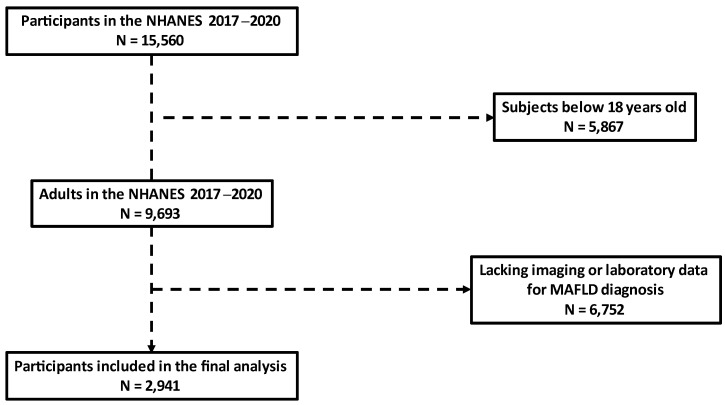
Flow chart of the study population inclusion process. NHANES: National Health and Nutrition Examination Survey. MAFLD: metabolic dysfunction–associated fatty liver disease.

Data were obtained from the NHANES between 2017 and March 2020 pre-pandemic for this study. NHANES databases are cross-sectional surveys conducted by the National Center for Health Statistics (NCHS), providing multitudinous health and nutrition data of the general United States population. A total of 15,560 individuals were enrolled in this study. After the exclusion of subjects below 18 years old (n = 5867) and those missing essential data for MAFLD diagnosis (n = 6752), 2941 participants were finally included in this analysis (Figure 1). This study was reviewed and approved by NCHS Ethics Review Board (protocol numbers #2011-17 and #2018-01). The patients/participants provided their written informed consent to participate in this study.

### 2.2. Assessment of Liver Steatosis and Fibrosis

Liver ultrasonography transient elastography was performed to non-invasively assess hepatic steatosis and fibrosis. Liver fibrosis was measured by FibroScan^®^, which uses ultrasound and the vibration-controlled transient elastography to derive liver stiffness. The device also simultaneously measures the ultrasound attenuation related to the presence of hepatic steatosis and records the controlled attenuation parameter (CAP) as the indicator for hepatic steatosis.

The elastography exam was performed by NHANES health technicians, who were trained and certified by NHANES staff and the equipment manufacturer (Echosens^TM^_,_ Waltham, MA, USA). The exams were performed according to the manufacturer’s guidelines. After selecting the correct wand and confirming that it is correctly positioned in the center of the liver, the health technicians took 10 valid measurements. If the first measurement was equal to or greater than 12 Kilopascals (KPa), the wand was repositioned and additional measurements were taken in at least three different sites. The health technicians then selected the site with the lowest stiffness value to obtain the 10 valid measurements. A measurement was considered valid when the shear wave propagation map was displayed with parallel margin shear waves and the interquartile range/median (IQR/M) was less than 30%. The IQR/M is recalculated after each new valid measurement. The median of all valid measurements was used to determine the degree of liver fibrosis, classified as F0 (<5.5 KPa), F1 (5.6–7.1 KPa), F2 (7.2–9.4 KPa), F3 (9.5–12.4 KPa), and F4 (≥12.5 KPa) [9]. Consequently, we categorized liver fibrosis as absent (F0), mild-moderate (F1-F2), or severe/cirrhosis (F3-F4). CAP measurement was recorded simultaneously with the liver stiffness measurement, and the median values were recorded.

Additionally, we evaluated the diagnostic role of the fatty liver index (FLI) in MAFLD. FLI is a well-established score regarding the presence of hepatic steatosis, especially if it exceeds 60 [10]. FLI was calculated according to the following formula: FLI = e^y^/(1 + e^y^) ∗ 100, where y = 0.953 ∗ ln(triglycerides in mg/dL) + 0.139 ∗ BMI in kg/m^2^ + 0.718 ∗ ln (gamma-glutamyl transferase (GGT) in U/L) + 0.053 ∗ waist circumference in cm − 15.745 [11]. Moreover, we assessed the diagnostic accuracy of FLI, Non-alcoholic fatty liver disease (NAFLD) fibrosis score (NFS), and fibrosis (FIB)4 score regarding the presence of significant fibrosis (F3-F4) on elastography. NFS was calculated according to the following formula: −1.675 + (0.037 ∗ age in years) + (0.094 ∗ BMI in kg/m^2^) + (1.13 ∗ impaired fasting glucose/diabetes [yes = 1, no = 0]) + (0.99 ∗ AST/ALT ratio) − (0.013 ∗ platelet count in cells × 10^9^/L) − (0.66 ∗ albumin in g/dL) [12]. FIB4 score was assessed through the following equation: (Age ∗ AST)/(Platelets ∗ √(ALT)) [13].

### 2.3. Definition of Variables

Individuals were stratified into two groups according to the presence or absence of MAFLD. The newly proposed criteria were applied [5]. Specifically, the presence of hepatic steatosis was confirmed in cases of median CAP values of ≥248 dB/m, as previously reported [14]. Accordingly, MAFLD was considered present in individuals with hepatic steatosis on liver elastography together with:

- Overweight/obesity (body mass index (BMI) ≥ 25 kg/m^2^ in Caucasians and ≥23 kg/m^2^ in Asians);
- T2DM (fasting plasma glucose ≥ 126 mg/dL, glycated hemoglobin ≥ 6.5% [15], or known history of the disease);
- At least two metabolic risk abnormalities:
  o Waist circumference ≥ 102/88 cm in Caucasian men and women or ≥90/80 cm in Asian men and women;
  o Blood pressure ≥ 130/85 mmHg or specific drug treatment;
  o Plasma triglycerides ≥ 150 mg/dL or specific drug treatment;
  o Plasma high-density lipoprotein cholesterol (HDL-C) < 40 mg/dL in men or <50 mg/dL in women, or specific drug treatment;
  o Prediabetes (fasting plasma glucose 100–125 mg/dL or glycated hemoglobin 5.7–6.4%);
  o Homeostasis model assessment of insulin resistance (HOMA-IR) ≥ 2.5;
  o High-sensitivity C reactive protein (hsCRP) > 2 mg/L.

Regarding the rest of the variables, waist circumference was assessed by placing a measuring tape in a horizontal plane around the abdomen at the level of the iliac crest. Blood pressure was measured with the Omron IntelliSense Blood Pressure Monitor (Model: HEM-907XL). During a 5-min resting period, the participant was instructed to sit with his/her back straight and supported, with both feet flat on the floor. The arm was supported, the upper arm was bare and unrestricted by clothing, and the cuff was placed at heart level. Three series of blood pressures were measured and the average measurements of systolic and diastolic blood pressure were recorded. Arterial hypertension was defined as systolic blood pressure ≥140 mmHg, diastolic blood pressure ≥90 mmHg, or the use of antihypertensive medication [16]. History of coronary artery disease, myocardial infarction, stroke, heart failure, chronic pulmonary disease, malignancy, and sleep disorders were self-reported. Renal function was assessed with the estimated glomerular filtration rate through the use of the 2021 CKD-EPI equation [17]. Chronic kidney disease (CKD) was considered in cases of eGFR < 60 mL/min/1.73 m^2^. The patient health questionnaire (PHQ)-9 was used as a metric of major depressive disorder, with the previously defined cutoff of ≥10 [18].

### 2.4. Laboratory Evaluations

The complete blood count was assessed by the Beckman Coulter DxH-800 Analyzer. The Roche Cobas 6000 analyzer was used to evaluate serum glucose, total cholesterol, HDL-C, triglycerides, hsCRP, alanine aminotransferase, aspartate aminotransferase, alkaline phosphatase, GGT, albumin, urea, creatinine, uric acid, and urinary creatinine. Urinary albumin was measured with the Fluorescein Immunoassay by SequoiaTurner Digital Fluorometer, Model 450. Serum ferritin concentration was derived by the electrochemiluminescence immunoassay “ECLIA”. Glycated hemoglobin and insulin were determined via the Tosoh G8 High-Performance Liquid Chromatography (HPLC) Glycohemoglobin Analyzer and the Tosoh AIA-900 Two-site Immunoenzymometric Assay, respectively. Insulin resistance was assessed according to the following formula: HOMA-IR = (Fasting insulin in uIU/mL) ∗ (Fasting glucose in mg/dL)/405 [19]. Low-density lipoprotein cholesterol (LDL-C) was estimated via the Friedewald equation: LDL-C = total cholesterol − HDL-C − (triglycerides/5) [20].

### 2.5. Statistical Analysis

Continuous variables were tested for normality through the Kolmogorov–Smirnov test and the visual inspection of P–P plots. All the examined variables did not follow a normal distribution, and are expressed as median (25th, 75th percentile). Categorical variables are presented as percentages. Between-group differences in continuous variables were assessed through the Mann–Whitney U nonparametric test. Differences between categorical variables were assessed by the formation of contingency tables and the performance of chi-square tests. Kruskal–Wallis one-way analysis of variance was used for the assessment of the differences of continuous variables between the fibrosis stages. Correlation analysis of nonparametric values was performed using the Spearman correlation coefficient. The diagnostic accuracy of FLI, NFS, and FIB4 was assessed through receiver operating characteristics (ROC) curve formation and calculation of the area under ROC curve (AUROC). The ideal cutoff values were chosen according to the Youden index [21], with subsequent assessment of its sensitivity and specificity. Cohen’s κ was run to determine if there was an agreement between the FLI-MAFLD definition and the elastography-MAFLD definition. All statistical calculations were performed in SPSS software (version 27.0; SPSS Inc., Chicago, IL, USA). All reported *p*-values were based on two-sided hypotheses, with a *p*-value of less than 0.05 being considered statistically significant.

## 3. Results

### 3.1. Epidemiology of MAFLD and Associations with Other Risk Factors

In this cross-sectional study of 2941 individuals, the prevalence of MAFLD was 58.6%. The sociodemographic characteristics and the medical history of the participants are presented in Table 1. Individuals with MAFLD were older, with higher obesity measures such as BMI and waist circumference. Interestingly, the prevalence of current smoking was lower in the MAFLD group (26.2% vs. 39.9%, *p* < 0.001). Individuals with MAFLD had significantly higher systolic and diastolic blood pressure compared to those without MAFLD. Regarding the patients’ medical history, the prevalence of arterial hypertension, T2DM, coronary artery disease, myocardial infarction, heart failure, and chronic pulmonary diseases was significantly higher in subjects with MAFLD. Moreover, MAFLD was characterized by more frequent sleep and depressive disorders, as well as malignancies. We did not observe any significant differences regarding the history of stroke and the presence of CKD.

### 3.2. Association of MAFLD with Paraclinical Biomarkers

We next examined the levels of various laboratory markers in the groups with and without MAFLD (Table 2). According to the results, a greater impairment was noted in markers of glucose and insulin homeostasis, renal function, liver function, as well as in the lipid profile of subjects with MAFLD. Moreover, individuals with MAFLD were characterized by a greater inflammatory burden as evidenced by the levels of hsCRP, hsCRP/albumin ratio, ferritin, and white blood cells.

### 3.3. The Role of Fatty Liver Index in MAFLD Diagnosis

**Figure 2:**
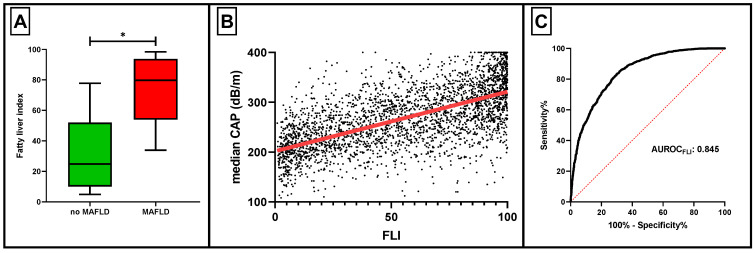
Fatty liver index (FLI) in metabolic dysfunction-associated fatty liver disease (MAFLD). (**A**) Box plots demonstrating the differences in FLI according to the presence of MAFLD. (**B**) Spearman correlation analysis of FLI with elastography-derived median controlled attenuation parameter (CAP). (**C**) Receiver operating characteristics (ROC) curve for the assessment of FLI’s diagnostic accuracy concerning MAFLD. AUROC: area under receiver operating characteristics curve. * indicates statistically significant difference (*p* < 0.05).

Since elastography or other imaging methods may not be available to all physicians for the estimation of steatosis, we examined whether FLI could represent an accurate diagnostic score regarding MAFLD. As expected, individuals with MAFLD had significantly higher values of FLI compared to those without MAFLD (Figure 2A). Moreover, a strong correlation was noted between FLI and median CAP (rho = 0.64, *p* < 0.001) (Figure 2B). According to the ROC curve analysis, FLI’s diagnostic performance was excellent (AUROC 0.845, *p* < 0.001) (Figure 2C). The ideal FLI cutoff for MAFLD diagnosis was determined to be ≥44.3, having a sensitivity and specificity of 83.6% and 69.7%, respectively. When the MAFLD definition according to a FLI ≥44.3 was set, there was moderate agreement with the elastography-derived MAFLD definition (κ = 0.542, *p* < 0.001).

### 3.4. Elastography-Derived Liver Fibrosis in MAFLD 

**Figure 3:**
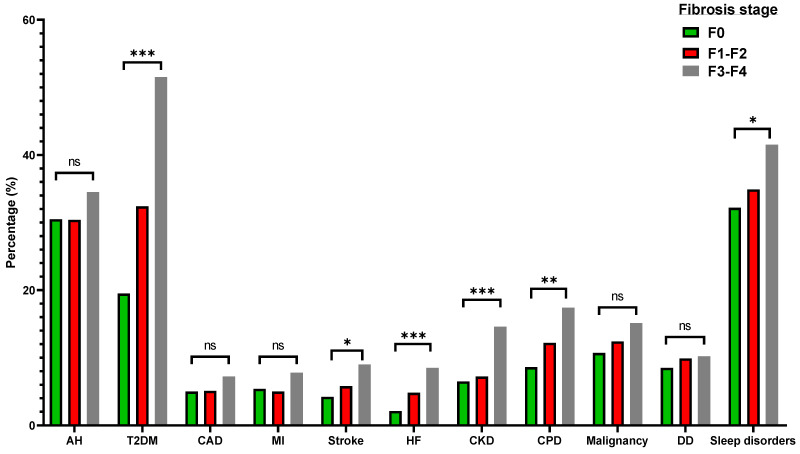
Differences in the prevalence of various pathologic states according to the fibrosis stage in individuals with metabolic dysfunction-associated fatty liver disease (MAFLD). AH: arterial hypertension. T2DM: type 2 diabetes mellitus. CAD: coronary artery disease. MI: myocardial infarction. HF: heart failure. CKD: chronic kidney disease. CPD: chronic pulmonary disease. DD: depressive disorder. ns indicates non-significant difference (*p* > 0.05), * *p* < 0.05, ** *p* < 0.01, *** *p* < 0.001.

In subjects with MAFLD, the prevalence of absent fibrosis (F0), mild-moderate fibrosis (F1-F2), and significant fibrosis-cirrhosis (F3-F4) was 54.4%, 35.7%, and 9.9%, respectively. We tried to determine the importance of elastography-derived liver fibrosis in individuals with MAFLD (Supplementary Table S1). As shown in Figure 3, there was a statistically significant, stepwise increase in the prevalence of T2DM, heart failure, stroke, chronic pulmonary disease, sleep disorders, and CKD, according to the degree of fibrosis. Even though a higher prevalence of arterial hypertension, coronary artery disease, myocardial infarction, and malignancies was observed in MAFLD individuals with severe fibrosis/cirrhosis, this did not reach statistical significance.

We finally assessed the importance of various established scores in identifying subjects with MAFLD and significant fibrosis (F3-F4). Between the FLI, the NFS, and the FIB4 score, FLI was the most accurate in this regard (Supplementary Figure S1). Specifically, it had the greatest diagnostic accuracy, with an AUROC of 0.781, followed by NFS (AUROC difference: −0.050, *p* = 0.04) and FIB4 score (AUROC difference −0.177, *p* < 0.001). The ideal cutoffs for the identification of significant fibrosis, along with their sensitivity and specificity, are presented in Table 3.

## 4. Discussion

In this analysis of the NHANES 2017–2020, the prevalence of MAFLD was extremely high, exceeding 50%. Moreover, individuals with MAFLD had a higher burden of cardiometabolic comorbidities such as arterial hypertension, T2DM, atherosclerotic cardiovascular diseases, and CKD. Moreover, they were more frequently affected by chronic pulmonary diseases, sleep disturbances, depression, and malignancies. Most of those comorbidities were increasingly more common in the presence of greater liver fibrosis. We additionally found that the group of MAFLD had significant abnormalities in markers of glucose homeostasis, renal and liver function, lipid profile, and inflammation. Finally, FLI emerged as a tool with excellent diagnostic accuracy regarding MAFLD with values ≥44.3, as well as an adequate ability to predict significant fibrosis at a higher cutoff (≥91). Interestingly, FLI’s diagnostic potential concerning fibrosis was significantly better than other established fibrosis scores, such as the NFS and the FIB4 score.

The prevalence of MAFLD in our study was 58.6%. Several reports have assessed the epidemiologic trends of MAFLD. Although some studies have demonstrated a lower prevalence (25–31.5%) [22,23,24,25], the study of Lee et al. pointed to a higher prevalence (37.3%) of MAFLD in a large health screening database [26]. In an analysis of the NHANES 2017–2018, MAFLD was present in 39.1% of the participants [27]. Interestingly, the global prevalence of MAFLD was estimated at 50.7% in a study using a meta-analytic approach in 2.667.052 individuals [28], similar to our observation. MAFLD was more prevalent in males [28], as in our study. The reported variations in the prevalence of MAFLD could be attributed to ethnic disparities, as studies with lower prevalence were conducted in Asian populations only [22,23,24,25,26]. Moreover, the differences in the methods of steatosis estimation (liver ultrasound, elastography, diagnostic scores) may account for some of the heterogeneity.

MAFLD may be associated with a higher burden of cardiovascular diseases and CKD, as shown in recently published studies following a longitudinal design. According to the study of Lee et al. in over 3 million individuals with MAFLD, the composite cardiovascular event occurred more frequently in the MAFLD group compared to the non-MAFLD group, especially in those with MAFLD and T2DM [29]. The increased cardiovascular risk and cardiovascular mortality rates were confirmed in subsequent meta-analyses [30,31]. CKD, sleep disturbances, and malignancies also occurred more frequently in individuals with MAFLD [30]. In our study, we also documented a higher prevalence of coronary artery disease, myocardial infarction, malignancies, and sleep disorders. However, we did not note an association with CKD, despite lower eGFR values in MAFLD individuals. These correlations with other diseases could be attributed to significant abnormalities in blood pressure, glucose–insulin homeostasis, impaired renal and kidney function, and dyslipidemia, as shown in our study. Moreover, inflammation could play a crucial role in the development and progression of MAFLD, as well as in the evolvement of hepatic and extrahepatic complications. Chronic, low-grade inflammation promotes oxidative stress and endothelial dysfunction [32,33,34], resulting in liver fibrosis, cirrhosis, and hepatocellular carcinoma [35]. Additionally, chronic inflammation may predispose to platelet activation [36] and, consequently, atherothrombosis [37]. At the level of the kidney, inflammation is also among the main pathogenetic mechanism of CKD development and progression [38].

Since the evaluation of hepatic steatosis, the cardinal feature of MAFLD, through the use of transient elastography may not be readily available, we tried to determine the potential importance of FLI in this regard. FLI is a score used to predict the presence of hepatic steatosis, incorporating waist circumference, BMI, triglycerides, and GGT. Its correlation with hepatic steatosis has been previously assessed [10]. In this analysis, we found an excellent accuracy of FLI to diagnose MAFLD, with a cutoff of ≥44.3. Accordingly, when we compared the prevalence of MAFLD according to median CAP ≥ 248 dB/m or FLI ≥ 44.3, a moderate agreement was reported. FLI has been previously assessed in a retrospective analysis of 1300 individuals undergoing computed tomography (CT). The diagnosis of MAFLD was set in cases of CT liver attenuation < 40 Hounsfield units or <10 Hounsfield units when compared to that of the spleen. The AUROC of FLI for MAFLD diagnosis was 0.791, and the ideal cutoff was ≥29.9. Han et al. reported similar results for FLI regarding CT-MAFLD (AUROC 0.776, cutoff ≥ 30.1) [39]. The use of a different index method for steatosis estimation in this study (CT) may have contributed to this disparity [40]. When using conventional liver ultrasonography for the establishment of steatosis, FLI’s AUROC is even lower (0.681), with a cutoff of ≥59.5 [41].

The evaluation of fibrosis is pivotal in individuals with liver disease, as it may predispose to cirrhosis and hepatocellular carcinoma development [42]. Moreover, it is frequently accompanied by an even higher burden of extrahepatic complications, namely cardiovascular and renal diseases [43,44,45]. In our study, we observed a higher prevalence of comorbidities in participants with increasing fibrosis stage, especially in F3-F4. Non-invasive fibrosis scores have not been adequately assessed in MAFLD [46]. Therefore, we analyzed the role of FLI, together with NFS and FIB4 score, in predicting F3-F4 fibrosis stage, and we found that FLI was superior to the latter scores. The established cutoff was extremely high, however (≥91). It should be noted that the existing fibrosis scores (NFS, FIB4) had a mediocre performance in this regard (AUROC_NFS_: 0.730, AUR_FIB4_: 0.605). Those predictive values were similar to that of biopsy-proven fibrosis in an Asian cohort (AUROC_NFS_: 0.699, AUR_FIB4_: 0.683). In another cohort of biopsy-proven MAFLD, NFS (AUROC: 0.724, cutoff ≥ 2.1) and FIB4 (AUROC: 0.736, cutoff ≥ 1.05) had decent diagnostic power [47].

Despite the large sample size and the use of transient elastography for the diagnosis of steatosis, our study has some limitations. First and foremost, the cross-sectional design does not allow for causal associations and the determination of MAFLD’s and diagnostic scores’ prognostic significance. Longitudinal and prospective cohort studies should be conducted to adequately assess this issue. Moreover, data collection for the period 2019–2020 was unavailable due to the coronavirus disease 2019 pandemic. Furthermore, the lack of a histological diagnosis of steatosis and fibrosis was not possible, and we cannot be sure of its possible influence on the study’s outcomes. Although transient elastography is highly sensitive and specific for liver fibrosis and steatosis, it is not the gold standard for their diagnosis [48]. Finally, the number of individuals being prescribed medications with proven benefits in fatty liver disease was extremely low in this study to be analyzed. Agents such as sodium-glucose cotransporter (SGLT)2 inhibitors or glucagon-like peptide (GLP)1 receptor agonists possess pleiotropic effects [49], including anti-inflammatory actions [50,51], and may lower the risk of incident MAFLD, as well as its complications [52].

## 5. Conclusions

Metabolic dysfunction-associated fatty liver disease is a highly prevalent pathologic condition that may be accompanied by numerous comorbidities. The presence of comorbidities may be increasingly common in individuals with higher grades of liver fibrosis. The estimation of fatty liver index could be a reasonable addition to the evaluation of patients with metabolic risk factors and could lead to a diagnosis in the absence of liver elastography or biopsy. Further studies are needed to enhance our knowledge regarding its prognosis, as well as the role of novel therapies in its prevention or regression.

## Figures and Tables

**Table 1:** Sociodemographic characteristics and medical history of the study population according to the presence of metabolic dysfunction-associated fatty liver disease (MAFLD).

	MAFLD (−) (N = 1217)	MAFLD (+) (N = 1724)	*p*
**Clinical characteristics**			
Age, years	46 (31, 62)	56 (43, 66)	<0.001
Male sex, %	53.0	55.9	0.13
BMI, kg/m^2^	25.1 (22.3, 28.4)	31.2 (27.2, 36.1)	<0.001
Waist circumference, cm	88.9 (80.6, 98.3)	106.5 (97.8, 117.8)	<0.001
Systolic blood pressure, mmHg	118 (109, 131)	124 (114, 137)	<0.001
Diastolic blood pressure, mmHg	71 (65, 78)	76 (69, 84)	<0.001
**Medical history**			
Arterial hypertension, %	19.8	30.9	<0.001
Type 2 diabetes mellitus, %	7.1	27.3	<0.001
Coronary artery disease, %	3.0	5.3	0.003
Myocardial infarction, %	2.9	5.5	<0.001
Stroke, %	4.1	5.2	0.15
Heart failure, %	2.2	3.7	0.02
Chronic kidney disease, %	6.1	7.5	0.13
Chronic pulmonary disease, %	6.9	10.8	<0.001
Malignancy, %	9.3	11.7	0.04
Sleep disorders, %	25.0	34.1	<0.001
Depressive disorder, %	7.2	9.2	0.05

**Table 2:** Differences in laboratory markers according to the presence of metabolic dysfunction-associated fatty liver disease (MAFLD).

	MAFLD (−) (N = 1217)	MAFLD (+) (N = 1724)	*p*
**Glucose-insulin homeostasis**			
Fasting plasma glucose, mg/dL	92 (86, 98)	101 (93, 117)	<0.001
Glycated hemoglobin (%)	5.4 (5.2, 5.7)	5.8 (5.4, 6.3)	<0.001
HOMA-IR	1.5 (1.0, 2.4)	3.6 (2.2, 5.9)	<0.001
**Renal function**			
eGFR, ml/min/1.73 m^2^	98.1 (82.4, 111.7)	95.6 (78.9, 108.2)	<0.001
Urinary albumin-to-creatinine ratio	6.6 (4.4, 11.0)	7.8 (5.0, 15.4)	<0.001
**Liver biochemistry**			
AST, IU/L	19 (16, 23)	20 (16, 25)	0.002
ALT, IU/L	15 (12, 22)	21 (15, 30)	<0.001
ALP, IU/L	71 (59, 86)	76 (64, 92)	<0.001
GGT, IU/L	18 (13, 26)	25 (18, 38)	<0.001
**Lipid profile**			
Total cholesterol, mg/dL	179 (155, 206)	183 (158, 213)	0.002
LDL-Cholesterol, mg/dL	103 (83, 126)	115 (93, 139)	<0.001
HDL-Cholesterol, mg/dL	56 (48, 66)	46 (40, 54)	<0.001
Triglycerides, mg/dL	84 (64, 114)	126 (90, 175)	<0.001
Uric acid, mg/dL	5.1 (4.3, 6.0)	5.7 (4.8, 6.8)	<0.001
**Inflammatory markers**			
hsCRP, mg/L	1.1 (0.6, 2.7)	2.6 (1.2, 5.2)	<0.001
hsCRP/albumin ratio	0.28 (0.13, 0.65)	0.65 (0.30, 1.35)	<0.001
Ferritin, ng/mL	103 (53, 185)	133 (65, 233)	<0.001
White blood cells (K, μL)	6000 (4900, 7200)	6700 (5600, 8200)	<0.001

HOMA-IR: homeostatic model assessment for insulin resistance. eGFR: estimated glomerular filtration rate. AST: aspartate aminotransferase. ALT: alanine aminotransferase. ALP: alkaline phosphatase. GGT: gamma-glutamyl transferase. LDL: low-density lipoprotein. HDL: high-density lipoprotein. hsCRP: high-sensitivity C reactive protein.

**Table 3:** Evaluation of AUROC curves and determination of cutoff values, together with sensitivity and specificity, for diagnostic scores regarding significant fibrosis in patients with metabolic dysfunction-associated fatty liver disease.

Score	AUROC Curve	95% Confidence Interval	*p*	Cutoff	Sensitivity	Specificity
FLI	0.781	0.75–0.82	<0.001	≥91.0	71.3	73.4
NFS	0.731	0.69–0.77	<0.001	≥−0.72	70.2	65.7
FIB4	0.605	0.56–0.65	<0.001	≥1.26	48.0	69.6

AUROC: area under receiver operating characteristics curve. FLI: fatty liver index. NFS: NAFLD fibrosis score. FIB4: fibrosis-4.

## Data Availability

Data are publicly available online (https://wwwn.cdc.gov/nchs/nhanes/Default.aspx, accessed on 13 October 2022). The datasets used and analyzed during the current study are available from the corresponding author upon reasonable request.

## Supplementary Materials

The following supporting information can be downloaded at: https://www.mdpi.com/article/10.3390/metabo12111070/s1, Figure S1: Receiver operating characteristics curve analysis of various diagnostic scores concerning significant fibrosis (F3-F4); Table S1: Sociodemographic characteristics and medical history of individuals with metabolic dysfunction-associated fatty liver disease according to the fibrosis stage.
