# Supplementary material for: Metabolic Dysfunction-Associated Fatty Liver Disease in the National Health and Nutrition Examination Survey 2017–2020: Epidemiology, Clinical Correlates, and the Role of Diagnostic Scores
Source: Metabolites. 2022 Nov 5;12(11):1070. doi: 10.3390/metabo12111070 (PMC9697527; doi:10.3390/metabo12111070)
Supplement: Supplementary file 1 [file metabolites-12-01070-s001.zip › metabolites-1999527-supplementary.pdf]

**Table S1.** Sociodemographic characteristics and medical history of individuals with metabolic dysfunction-associated fatty liver disease according to the fibrosis stage.

| | F0 (N=938) | F1-2 (N=615) | F3-4 (N=171) | p |
|---|---|---|---|---|
| **Sociodemographic characteristics** | | | | |
| Age, years | 56 (42, 65) | 54 (41, 65) | 60 (48, 68)*,† | 0.009 |
| Male sex, (%) | 50.9 | 62.9* | 57.9 | <0.001 |
| BMI, $kg/m^2$ | 30.0 (26.8, 34.2) | 32.4 (28.7, 37.2)* | 36.9 (31.7, 45.4)*,† | <0.001 |
| Waist circumference, cm | 102.7 (94.6, 112.7) | 109.5 (101.0, 120.8)* | 121.3 (110.5, 133.8)*,† | <0.001 |
| Systolic blood pressure, mmHg | 123 (112, 136) | 126 (116, 137)* | 126 (116, 140) | 0.02 |
| Diastolic blood pressure, mmHg | 75 (68, 83) | 77 (69, 85)* | 76 (68, 84) | 0.01 |
| **Medical history** | | | | |
| Arterial hypertension (%) | 30.5 | 30.4 | 34.5 | 0.55 |
| Type 2 diabetes mellitus (%) | 19.5 | 32.4* | 51.5*,† | <0.001 |
| Coronary artery disease (%) | 5.0 | 5.1 | 7.2 | 0.50 |
| Myocardial infarction (%) | 5.4 | 5 | 7.8 | 0.36 |
| Stroke (%) | 4.2 | 5.8 | 9.0* | 0.03 |
| Heart failure (%) | 2.1 | 4.8* | 8.5* | <0.001 |
| Chronic kidney disease (%) | 6.5 | 7.2 | 14.6* | <0.001 |
| Chronic pulmonary disease (%) | 8.6 | 12.2 | 17.4* | 0.001 |
| Malignancy (%) | 10.7 | 12.4 | 15.1 | 0.23 |
| Sleep disorders (%) | 32.2 | 34.9 | 41.5* | 0.05 |
| Depressive disorder (%) | 8.5 | 9.9 | 10.2 | 0.59 |

* indicates statistically significant difference (p<0.05) vs. F0

† indicates statistically significant difference (p<0.05) vs. F1-F2

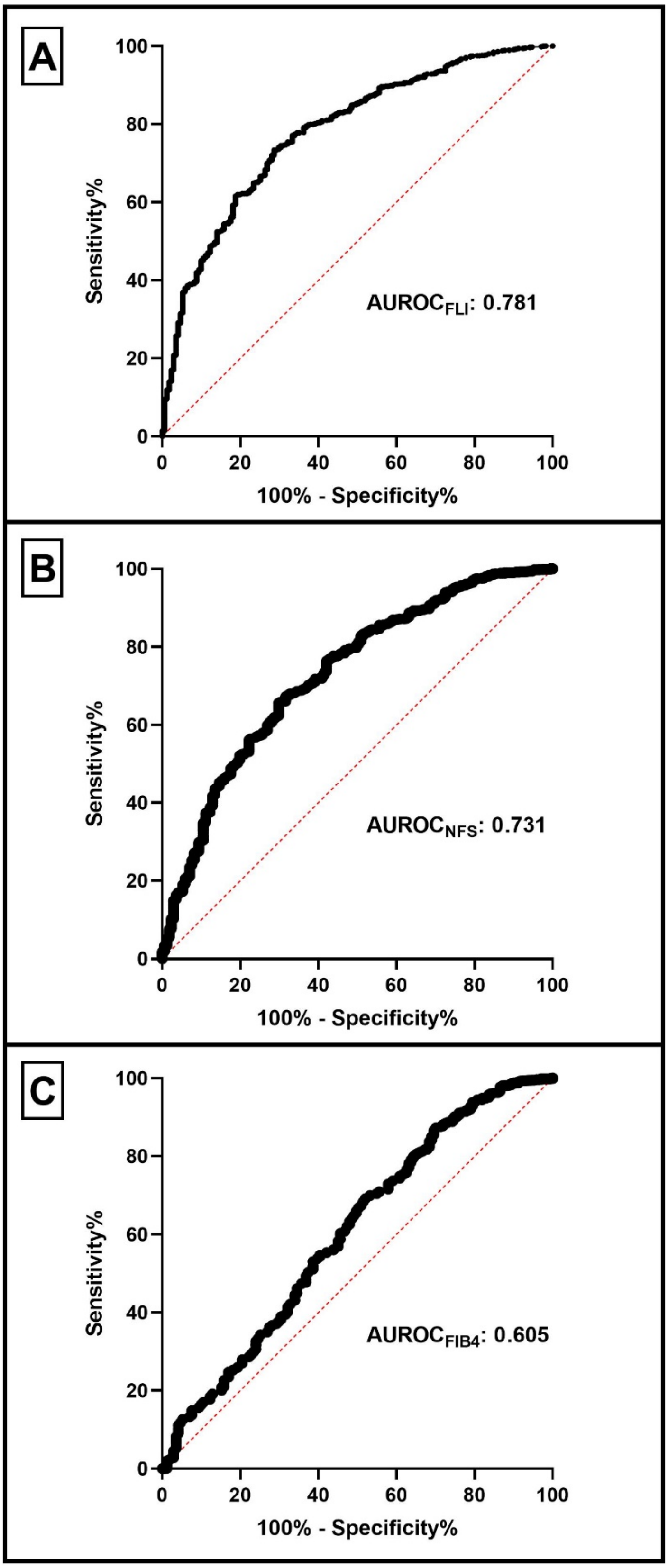


**Figure S1**. Receiver operating characteristics curve analysis of various diagnostic scores concerning significant fibrosis (F3-F4).
